# SMARCA2 deficiency in NSCLC: a clinicopathologic and immunohistochemical analysis of a large series from a single institution

**DOI:** 10.1265/ehpm.21-00254

**Published:** 2022-02-19

**Authors:** Shanshan Sun, Qiujing Li, Zhenkun Zhang, Sili Xiong, Yujie Zhang, Qian Liu, Zhe Li, Fujun Yang, Shukun Zhang

**Affiliations:** 1 Department of Oncology, Weihai Municipal Hospital, Cheeloo College of Medicine, Shandong University, Weihai, Shandong, 264200, China; 2 Department of Pathology, Weihai Municipal Hospital, Cheeloo College of Medicine, Shandong University, Weihai, Shandong, 264200, China; 3 Department of Oncology, Shouguang People’s Hospital, Weifang, Shandong, 262700, China; 4 Weifang Medical College, Weifang, Shandong, 261053, China

**Keywords:** SWI/SNF, SMARCA2, NSCLC, Tissue microarray, PD-L1, Prognosis

## Abstract

**Background:**

SMARCA2 (SWI/SNF Related, Matrix Associated, Actin Dependent Regulator of Chromatin, Subfamily A, Member 2) is an important ATPase catalytic subunit in the switch-sucrose nonfermenting (SWI/SNF) complex. However, its relationship with the pathological features of NSCLC and its prognosis remain unclear.

**Methods:**

We retrospectively reviewed 2390 patients with surgically resected NSCLC, constructed tissue microarrays (TMAs) and performed immunohistochemical assays. We analyzed the correlation of SAMRCA2 with clinicopathological features and evaluated its prognostic value.

**Results:**

Among 2390 NSCLC cases, the negative expression ratios of SAMRCA2, SMARCA4, ARID1A, ARID1B and INI1 were 9.3%, 1.8%, 1.2%, 0.4% and 0%, respectively. In NSCLC, male sex, T3 and T4 stage, moderate and poor differentiation, tumor ≥ 2 cm, Ki67 ≥ 15%, SOX-2 negative expression, middle lobe lesion and adenocarcinoma were relative risk factors affecting SMARCA2-negative expression. In lung adenocarcinomas, high-grade nuclei, histological morphology of acinar and papillary, solid and micropapillary and TTF-1-negative expression were relative risk factors affecting SMARCA2-negative expression. Kaplan–Meier survival analysis showed that the OS was shorter in the SMARCA2-negative group. Multivariate survival analysis revealed that SMARCA2-negative expression was an independent factor correlated with a poor prognosis in NSCLC.

**Conclusion:**

In conclusion, SMARCA2-negative expression is an independent predictor of a poor outcome of NSCLC and is a potential target for NSCLC treatment.

## 1. Introduction

According to Global Cancer Statistics 2020, lung cancer is now the second most common malignancy among new cancer cases worldwide (11.4% of all new cases), and it remains the “leading cause” of cancer death (18.0% of all cancer deaths) [1]. In China, lung cancer is the most common malignant tumor, with the highest morbidity and mortality from cancer in all populations [2]. Although the morbidity and mortality of lung cancer have decreased slightly in recent years with the continuous improvement of diagnostic and treatment techniques, the 5-year survival rate of lung cancer patients is still not optimistic. In 2000, the 5-year survival rate of lung cancer in China was 18.6%, and in 2010, there was an upward trend but it was still less than 20% [3]. Its highly heterogeneous nature and the high rate of recurrence and metastasis make the long-term treatment outcomes of lung cancer unsatisfactory. Therefore, it is important to find new potential therapeutic targets and prognostic indicators.

Cellular DNA is wrapped in an octamer of histones to form nucleosomes, the basic structural units of chromatin. The adjacent nucleosomes in chromatin gradually curl up to form dense heterochromatin, blocking cellular activities such as gene transcription and replication. Chromatin remodeling loosens the dense structure of chromatin and exposes the DNA in the nucleosomes, promoting the recruitment of transcriptional regulators, gene transcription, replication and DNA repair. Two main mechanisms of chromatin remodeling exist, including ATP-dependent physical modification and chemical modifications of chromatin. SWI/SNF is a highly evolutionarily conserved multisubunit protein complex that uses energy generated by ATP hydrolysis to accomplish chromatin remodeling, prompting gene transcriptional regulation, replication and DNA repair.

SWI/SNF mainly relies on catalytic subunits for its function. Genetic mutations of subunits of the SWI/SNF complex lead to tumor progression in a variety of tumors, including rhabdoid tumors [4], SCCOHT [5], gastric cancer [6], and hepatocellular carcinoma [7]. However, it has also been found that different subunits of this complex are overexpressed in various types of tumors [8]. The same protein shows different expression statuses and plays different roles in different stages of tumors [9]. Although the specific mechanism of these phenomena is still unclear, the SWI/SNF complex plays an important role in the occurrence and development of the tumor. A variety of small molecule drugs targeting the SWI/SNF complex and its related signaling pathways have emerged [10], some of which have shown good antitumor effects [11]. Therefore, it is of great significance to accurately identify the expression status of SWI/SNF in clinical practice. Additionally, the patients can be stratified according to the expression status of SWI/SNF to guide clinicians toward more targeted antitumor therapy.

The SMARCA2 gene is a very important catalytic subunit in the SWI/SNF complex, essential for SWI/SNF activities, such as cellular metabolism (including drug metabolism), differentiation, development, DNA repair, tumor angiogenesis, progression or metastasis [12]. Although SMARCA2 has been found in a wide range of human cancers, the importance of the altered expression or dysfunction of the SMARCA2 protein in various cancers is not fully understood. To date, only a few studies have evaluated the relationship between SMARCA2 and clinicopathological factors in NSCLC. In this study, for the first time, we screened data from a large clinical cohort to investigate the expression of SMARCA2 protein in NSCLC. Additionally, we analyzed the correlation between SMARCA2 expression and the clinicopathological characteristics and prognosis in NSCLC. We aimed to provide a clinical basis for further exploration of SMARCA2 as a potential therapeutic target and for the establishment of a prognostic assessment system in NSCLC.

## 2. Materials and methods

### 2.1 Clinical cases selection

We screened cases treated with surgical resection for lung cancer at Weihai Municipal Hospital from January 2014 to December 2020 and collected cases with pathological tissue diagnosed as NSCLC. The exclusion criteria were as follows: 1). carcinoma in situ; 2). metastatic lung cancer and 3). surgery after neoadjuvant therapy. This study was approved by the Ethics Committee of Weihai Municipal Hospital, Shandong University.

### 2.2 Review of whole sections of tumor tissue from the selected cases

Tissue specimens were fixed in 10% formalin and embedded in paraffin, and the dewaxed sections were stained with hematoxylin-eosin. Two pathologists reviewed all original tissue sections of all screened cases to determine whether the screened cases met the inclusion criteria, and if not, they were excluded.

### 2.3 Construction of tumor TMAs (tissue microarrays)

The sections were carefully reviewed, and representative tumor regions were selected as regions of interest for the study. Markers were made on HE section slides for subsequent preparation of tissue microarrays by punching the corresponding tissue wax blocks. Tissue microarrays were prepared with a manual tissue microarray sampling gun (JLM-5113, Guangdong, China). The tissue columns were incorporated into 60-well TMA wax molds and paraffin-embedded. Then, the sections of the TMA (2 µm) were transferred to detachable slides, and HE staining was performed by applying an automatic stainer (Ventana HE600, Roche, USA).

### 2.4 Pathologic studies for SWI/SNF (antibodies and immunohistochemical staining)

Immunohistochemical staining was performed using an automated immunostainer (Benchmark ULTRA, Ventana). The antibodies against the following proteins were: SMARCA2 (EPR23103-44, Abcam, 1:400), ARID1A (EPR13501, Abcam, 1:1000), ARID1B (2D2, Abcam, 1:500), BRG1 (E8V5B, Origene, Ready-to-use), INI1 (25, Origene, Ready-to-use), Napsin-A (IP64, Origene, Ready-to-use), SOX-2 (EP103, Origene, Ready-to-use), P53 (DO-7, Ibp, Ready-to-use), TTF-1 (SPT24, Ibp, Ready-to-use), Ki-67(MyM1-Ki67, Ibp, Ready-to-use), ALK (D5F3, Ventana, Roche), and PD-L1 (SP263, Ventana, Roche).

### 2.5 Immunohistochemical staining assessment

Immunohistochemical evaluation criteria of the SWI/SNF complex: 1). Five levels according to the percentage of positive cell staining: 0 for <1%, 1 for 1%–25%, 2 for 26%–50%, 3 for 51%–75%, and 4 for >75%; 2). Four levels were used according to the intensity of positive cell nuclei staining: 0 for no staining, 1 for light yellow, 2 for yellow, and 3 for brownish-yellow. Each tissue in the microarray corresponded to a percentage number and a color intensity score, and the product of the two scores was the final score. In our study, a final score of 0 was defined as negative expression, and the rest were defined as positive expression. Ventana-SP263 IHC was applied for PD-L1 detection, and PD-L1 expression was assessed according to the TPS scoring system, classified as ≤1%, 1%–50%, and ≥ 50%. Ventana-D5F3 IHC was used for ALK detection, the staining was localized in the cytoplasm, and those with strong positive staining were considered ALK-positive. For cases with uncertain staining, FISH was performed to verify the staining. P53 wild-type expression was defined as less than 80% of tumor cells showing different intensities of P53 nuclear staining; P53 aberrant expression was shown by a complete absence of P53 nuclear staining in tumor cells, and >80% of tumor cells showed strong positive P53 nuclear staining.

### 2.6 Clinicopathological features collection

Age, sex, history of underlying lung disease, smoking history, family history, tumor location, tumor size, lymphatic metastasis, distant metastasis, pleural invasion, vascular thrombosis, airway dissemination, necrosis, degree of tumor differentiation, nuclear grade, cytoplasmic eosinophilic status, pathological stage (the 8^th^ edition of the TNM classification), and pathological histological type (the 5th edition of the WHO) were collected. A total of 569 cases of NSCLC with EGFR detection (ARMS-PCR) were selected for analysis. The final valid cases were selected for the correlation analysis with clinicopathological characteristics because of tissue loss on TMA during immunohistochemical testing. Survival data were obtained by searching inpatient medical records and telephone follow-up.

### 2.7 Statistical analysis

All statistical analyses were performed using R language (version 3.6.1). First, the correlation between SMARCA2 expression and the clinicopathological characteristics was analyzed using Pearson χ2 correlation analysis. Then, the factors with P < 0.05 in the univariate chi-square test were selected for the multivariate analysis of logical regression. Overall survival (OS) time was defined as the time from the date of surgery to the date of death or the last follow-up. Progression-free survival (PFS) time was defined as the time from the date of surgery to the date of tumor progression or the last follow-up at which no progression was observed. Survival curves were analyzed using Kaplan–Meier analysis, and statistical significance was evaluated using the log-rank test. Risk assessment of SMARCA2 negative expression in OS and PFS of NSCLC used Cox univariate and multifactorial regression analyses. P < 0.05 was considered a statistically significant difference.

## 3. Results

### 3.1 Negative expression of the SWI/SNF complex in NSCLC

3.1.1 Based on the criteria mentioned above, 2390 cases (among 2609) were finally included in the study. The proportions of lung cancer tissue types in the study cohort were lung adenocarcinoma (nonmucinous) (2084 cases, 87.2%); mucinous adenocarcinoma (90 cases, 3.8%); squamous cell carcinoma (200 cases, 8.3%); and others (16 cases, 0.7%), including 12 cases of large cell neuroendocrine carcinoma, 3 cases of large cell carcinoma and 1 case of spindle cell carcinoma.

**Fig. 1:**
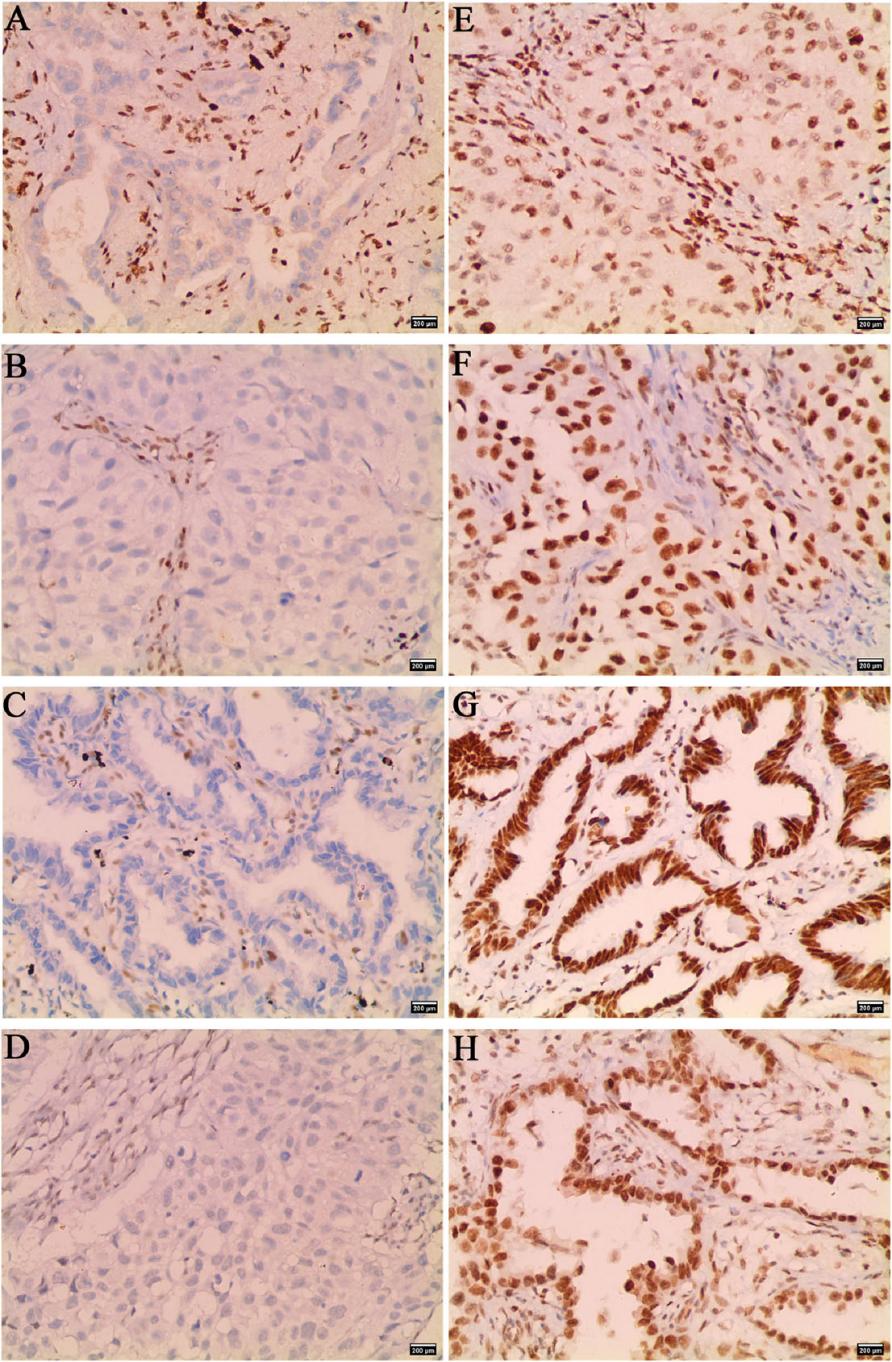
Immunohistochemical staining images of negative and positive expression of SMARCA2, SMARCA4, ARID1A, and ARID1B in NSCLC. Negative expression of (**A**) SMARCA2, (**B**) SMARCA4, (**C**) ARID1A, (**D**) ARID1B. Positive expression of (**E**) SMARCA2, (**F**) SMARCA4, (**G**) ARID1A, (**H**) ARID1B (×200).

3.1.2 Positive staining of the SWI/SNF subunits was localized in the nucleus, while no nuclear staining was observed in NSCLC cells with SWI/SNF subunit deletion, and as an internal control, the nuclei of infiltrating lymphocytes or bronchial epithelial cells in the same section were positive (Fig. 1).

3.1.3 According to the evaluation of the tissue microarrays, the negative expression rates of the five subunits of the SWI/SNF complex in NSCLC were 9.3% (9.6% of 2084 nonmucinous adenocarcinomas, 5.6% of 90 mucinous adenocarcinomas, 6.0% of 200 squamous cell carcinomas, and 31.3% of 16 others) for SMARCA2, 1.8% (1.5% of 2079 nonmucinous adenocarcinomas, 6.7% of 89 mucinous adenocarcinomas, 2.0% of 200 squamous cell carcinomas, and 0% of 16 others) for SMARCA4, 1.2% (0.8% of 2081 nonmucinous adenocarcinomas, 4.5% of 89 mucinous-adenocarcinomas, 4.5% of 199 squamous cell carcinomas, and 0% of 16 others) for ARID1A, and 0.4% (0.3% of 2080 nonmucinous adenocarcinomas, 0% of 90 mucinous-adenocarcinomas, 1.0% of 200 squamous cell carcinomas, and 0% of 16 others) for ARID1B. No negative expression of INI1 was found in any of the NSCLC cases (Table 1).

**Table 1:** The negative expression ratios of five subunits in the SWI/SNF complex in 2390 cases with NSCLC.

**Histology**	**The proportion of negative expression of SWI/SNF complex subunits**									

	**SMARCA2**		**SMARCA4**		**ARID1A**		**ARID1B**		**INI1**	
AD(Nonmucinous)	n = 2084	200(9.6%)	n = 2079	32(1.5%)	n = 2081	16(0.8%)	n = 2080	7(0.3%)	n = 2077	0(0)
AD(Mucinous)	n = 90	5(5.6%)	n = 89	6(6.7%)	n = 89	4(4.5%)	n = 90	0(0)	n = 89	0(0)
SCC	n = 200	12(6%)	n = 200	4(2.0%)	n = 199	9(4.5%)	n = 200	2(1.0%)	n = 200	0(0)
Others	n = 16	5(31.3%)	n = 16	0(0)	n = 16	0(0)	n = 16	0(0)	n = 16	0(0)

Note: AD, adenocarcinoma; SCC, squamous cell carcinoma.

3.1.4 A codeficiency of two or more subunits of the SWI/SNF complex was observed in 1.2% (29/2390) of NSCLC cases: 7 cases for SMARCA2 and SMARCA4; 6 cases for SMARCA2 and ARID1A; 3 cases for SMARCA2 and ARID1B; 3 cases for SMARCA4 and ARID1A; 7 cases for SMARCA2, SMARCA4 and ARID1A; 2 cases for SMARCA2, SMARCA4 and ARID1B; and 1 case for SMARCA2, SMARCA4, ARID1A and ARID1B (Table 2).

**Table 2:** Co-deficiency expression of SWI/SNF complex subunits in 2390 cases with NSCLC.

**Category**	**Co-deficiency of SWI/SNF** **complex subunits**	**n** **(total = 29)**
2 subunits	SMARCA2+SMARCA4	7
	SMARCA2+ARID1A	6
	SMARCA2+ARID1B	3
	SMARCA4+ARID1A	3
3 subunits	SMARCA2+SMARCA4+ARID1A	7
	SMARCA2+SMARCA4+ARID1B	2
4 subunits	SMARCA2+SMARCA4+ARID1A+ARID1B	1

### 3.2 Analysis of clinicopathological features related to the negative expression of SMARCA2 in NSCLC

**Fig. 2:**
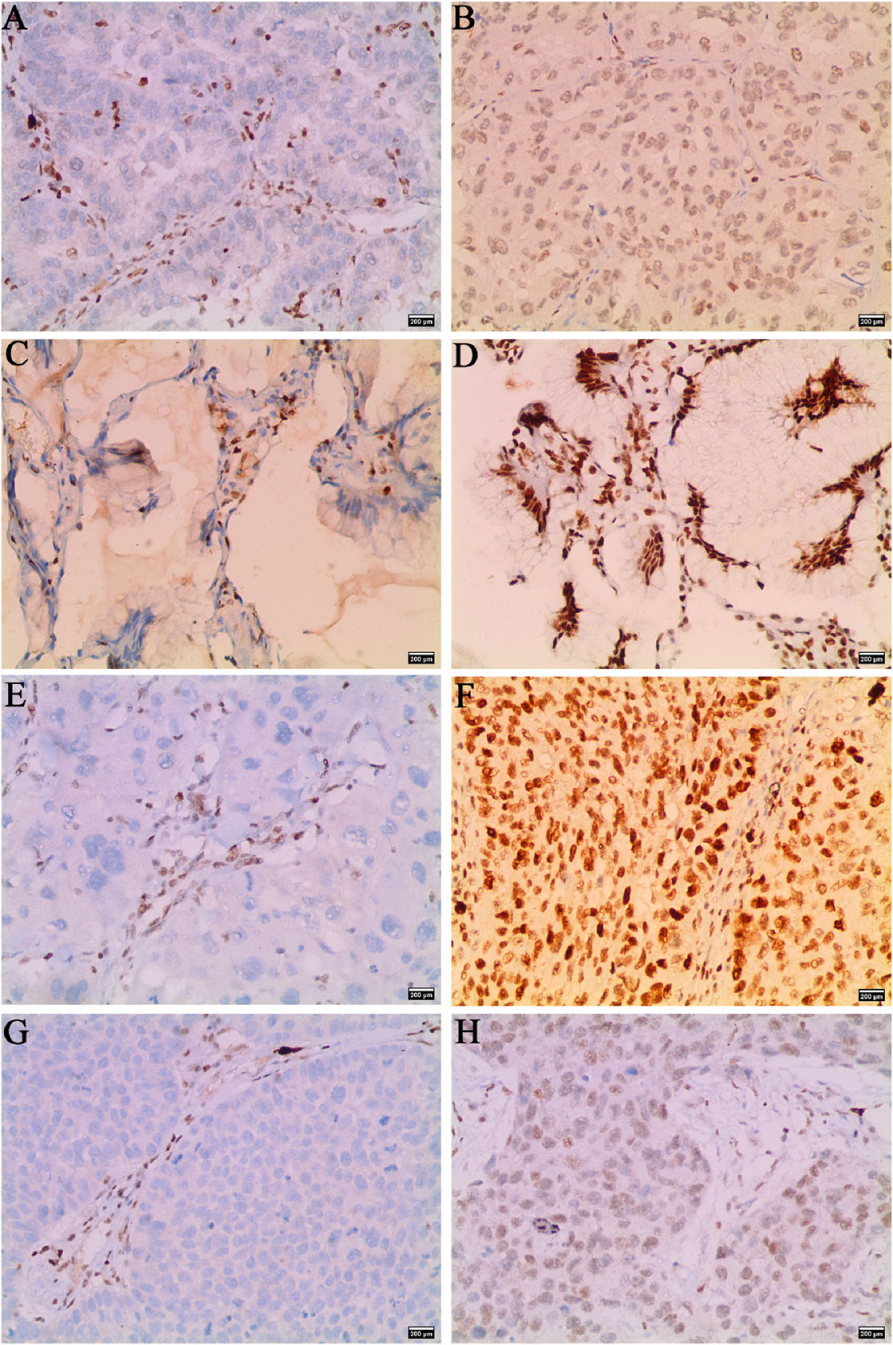
Immunohistochemical staining images of negative and positive SMARCA2 expression in non-mucinous AD, mucinous AD, SCC, and LCC. (**A**, **B**) for SMARCA2 negative and positive expression in non-mucinous AD, (**C**, **D**) for SMARCA2 negative and positive expression in mucinous AD, (**E**, **F**) for SMARCA2 negative and positive expression in SCC, (**G**, **H**) for SMARCA2 negative and positive expression in LCC (×200).

3.2.1 In lung cancer tumor tissue, SMARCA2-positive staining was localized in the nuclei of tumor cells, whereas SMARCA2-negative expression was absent in the nuclei of NSCLC cells. In the nonneoplastic lung parenchyma, SMARCA2 was expressed in the nuclei of bronchial epithelial cells, alveolar wall cells and inflammatory cells, which could serve as positive controls for the same sections (Fig. 2).

3.2.2 The results of univariate analysis of factors related to SMARCA2-negative expression are shown in Table 3. Compared to SMARCA2-positive expression, SMARCA2-negative expression was significantly associated with male sex, a larger tumor size, higher T stage, lymphatic metastasis, higher pathologic stage, pleural invasion, vascular invasion, necrosis, poor differentiation, higher proliferation index, P53 aberrant expression, high PD-L1 expression, and SOX-2-negative expression, and the proportion of SMARCA2-negative expression was higher in the middle lobe lesions than in the other lung lobes (P < 0.05). Other clinicopathological variables, including age, smoking history, emphysema or tuberculosis, family history, distant metastasis, STAS, EGFR mutation, and ALK mutation, showed no correlation with SMARCA2 expression.

**Table 3:** Correlation of SMARCA2 negative expression with clinicopathological features in 2390 cases with NSCLC.

	**Total n (%)**	**Expression of SMARCA2**		**P-value**	**χ^2^**

		**Positive n (%)**	**Negative n (%)**		
Age-years	2390	2168	222		
≤60	1018(42.6)	930(42.9)	88(39.6)	0.349	0.874
>60	1372(57.4)	1238(57.1)	134(60.4)		
Gender	2390	2168	222		
Male	1077(45.1)	952(43.9)	125(56.3)	4.076E-04^*^	12.497
Female	1313(54.9)	1216(56.1)	97(43.7)		
Smoking history	2390	2168	222		
Yes	568(23.8)	507(23.4)	61(27.5)	0.173	1.861
No	1822(76.2)	1661(76.6)	161(72.5)		
Emphysema/Tuberculosis	2390	2168	222		
Yes	77(3.2)	66(3.0)	11(95.0)	0.125	2.358
No	2313(96.8)	2102(97.0)	211(5.0)		
Family history	2390	2168	222		
Yes	295(12.3)	267(12.3)	28(12.6)	0.898	0.016
No	2095(87.7)	1901(87.7)	194(87.4)		
Tumor location	2390	2168	222		
Central	160(6.7)	146(6.7)	14(6.3)	0.808	0.059
Peripheral	2230(93.3)	2022(93.3)	208(93.7)		
Different lung lobes	2390	2168	222		
Middle lobe	196(8.2)	169(7.8)	27(12.2)	0.024^*^	5.101
Upper & lower lobe	2194(91.8)	1999(92.2)	195(87.8)		
Tumor size	2390	2168	222		
<2 cm	1125(47.1)	1064(49.1)	61(27.5)	8.201E-10^*^	37.712
≥2 cm	1265(52.9)	1104(50.9)	161(72.5)		
T-stage	2390	2168	222		
T1	1627(68.1)	1514(69.8)	113(50.9)	1.465E-10^*^	48.763
T2	647(27.1)	565(26.1)	82(36.9)		
T3	67(2.8)	49(2.3)	18(8.1)		
T4	49(2.0)	40(1.8)	9(4.1)		
N-stage	2390	2168	222		
N0	2007(84.0)	1849(85.3)	158(71.2)	4.757E-08^*^	29.814
N+	383(16.0)	319(14.7)	64(28.8)		
M-stage	2390	2168	222		
M0	2371(99.2)	2151(99.2)	220(99.1)	1.000	2.983E-28
M+	19(0.8)	17(0.8)	2(0.9)		
TNM Stage (UICC/AJCC 8th)	2390	2168	222		
I	1897(79.4)	1758(81.1)	139(62.6)	2.556E-09^*^	42.923
II	194(8.1)	161(7.4)	33(14.9)		
III	280(11.7)	232(10.7)	48(21.6)		
IV	19(0.8)	17(0.8)	2(0.9)		
Pleural invasion	2390	2168	222		
Yes	523(21.9)	449(20.7)	74(33.3)	1.474E-05^*^	18.771
No	1867(78.1)	1719(79.3)	148(66.7)		
Vascular invasion	2390	2168	222		
Yes	120(5.0)	98(4.5)	22(9.9)	4.612E-04^*^	12.266
No	2270(95)	2070(95.5)	200(90.1)		
STAS	2390	2168	222		
Yes	155(6.5)	141(6.5)	14(6.3)	0.909	0.013
No	2235(93.5)	2027(93.5)	208(93.7)		
Necrosis	2390	2168	222		
Yes	228(9.5)	189(8.7)	39(17.6)	1.911E-05^*^	18.276
No	2162(90.5)	1979(91.3)	183(82.4)		
Differentiation states	2386	2164	222		
Well	592(24.8)	577(26.7)	15(6.8)	<2.200E-16^*^	84.030
moderate	923(38.7)	856(39.5)	67(30.2)		
Poor	871(36.5)	731(33.8)	140(63.0)		
Ki-67	2389	2167	222		
<15%	1177(49.3)	1114(51.4)	63(28.4)	6.294E-11^*^	42.727
≥15%	1212(50.7)	1053(48.6)	159(71.6)		
P53	2388	2166	222		
Aberrant	720(30.2)	632(29.2)	88(39.6)	1.217E-03^*^	10.464
WT	1668(69.8)	1534(70.8)	134(60.4)		
SOX-2	2390	2168	222		
Positive	683(28.6)	638(29.4)	45(20.3)	0.004^*^	8.274
Negative	1707(71.4)	1530(70.6)	177(79.7)		
PD-L1	2386	2164	222		
≤1%	1987(83.3)	1818(84.0)	169(76.1)	0.003^*^	11.633
1%–50%	186(7.8)	166(7.7)	20(9.0)		
≥50%	213(8.9)	180(8.3)	33(14.9)		
EGFR	569	504	65		
MT	270(47.5)	237(47.0)	33(50.8)	0.569	0.324
WT	299(52.5)	267(53.0)	32(49.2)		
ALK	2390	2168	222		
Positive	40(1.7)	37(1.7)	3(1.4)	0.906	0.014
Negative	2350(98.3)	2131(98.3)	219(98.6)		
Classification	2390	2168	222		
AD	2174(91.0)	1969(90.8)	205(92.3)	0.003^*^	11.777
SCC	200(8.4)	188(8.7)	12(5.4)		
Others	16(0.6)	11(0.5)	5(2.3)		

Note: ^*^P < 0.05; STAS: spread through air spaces; WT: Wild type; MT: Mutant type.

Factors that were significantly different by univariate chi-square test were included in the multifactor logistic regression analysis to derive independent factors correlated with SMARCA2-negative expression. The results of the multifactor logistic regression analysis are shown in Table 4, which showed that sex, cancer type, T stage, differentiation states, tumor size, proliferation index, SOX-2, and lung lobes were the influencing factors of SMARCA2-negative expression. In detail, male sex, T3 and T4 stage, moderate and poor differentiation, tumor ≥ 2 cm, Ki67 ≥ 15%, SOX-2 negative expression, middle lobe lesion and adenocarcinoma were relative risk factors affecting SMARCA2-negative expression (P < 0.05).

**Table 4:** Factors associated with SMARCA2-negative expression in NSCLC patients.

**Factors**	**OR**	**95% Confidence interval**	**P-value**
Gender			
Female	1.000		
Male	1.452	1.072–1.968	0.016^*^
T-stage			
T1	1.000		
T2	1.061	0.755–1.486	0.731
T3	3.069	1.564–5.881	8.574E-04^*^
T4	2.495	1.036–5.564	0.031^*^
Tumor location			
Upper & lower lobe	1.000		
Middle lobe	2.022	1.257–3.218	2.623E-03^*^
Differentiation states			
Well	1.000		
moderate	2.772	1.588–5.143	6.150e-04^*^
Poor	4.965	2.791–9.374	1.830E-07^*^
Tumor size			
<2 cm	1.000		
≥2 cm	1.848	1.310–2.626	5.304E-04^*^
Classification			
SCC	1.000		
AD	3.413	1.766–7.115	5.065E-04^*^
Others	6.931	1.813–24.278	2.959E-03^*^
Ki67			
<15%	1.000		
≥15%	1.457	1.014–2.105	0.043^*^
SOX-2			
Positive	1.000		
Negative	2.586	1.774–3.855	1.539E-06^*^

### 3.3 Analysis of cell characteristics affecting SMARCA2-negative expression in lung adenocarcinoma

The correlation between SMARCA2 expression and cytological features in lung adenocarcinoma is shown in Table 5. In 2174 lung adenocarcinomas, SMARCA2-negative expression (n = 205) was significantly correlated with high-grade nuclei, eosinophilic staining of the cytoplasm, solid and micropapillary histological subtypes, TTF-1-negative expression and Napsin-A-negative expression (P < 0.05). The results of the logistic regression (Table 6) showed that nuclear grade, histological subtype, and TTF-1 were factors influencing SMARCA2-negative expression. Among them, high-grade nuclei, acinar and papillary histological morphology, solid and micropapillary morphology and TTF-1-negative status were relative risk factors affecting SMARCA2-negative expression. (P < 0.05).

**Table 5:** Correlation of SMARCA2-negative expression with cytological features in lung adenocarcinoma.

	**Total n (%)**	**Expression of SMARCA2**		**P-value**	**χ^2^**

		**Positive n (%)**	**Negative n (%)**		
Nuclear grade	2170	1965	205		
Low	191(8.8)	185(9.4)	6(2.9)	3.074E-14^*^	62.226
Intermediate	1283(59.1)	1199(61.0)	84(41.0)		
High	696(32.1)	581(29.6)	115(56.1)		
Eosinophilic staining of cytoplasm	2170	1965	205		
Yes	418(19.3)	351(17.9)	67(32.7)	3.051E-07^*^	26.217
No	1752(80.7)	1614(82.1)	138(67.3)		
Histological	2170	1965	205		
Lepidic	589(27.1)	574(29.2)	15(7.3)	1.165E-22^*^	101.010
Acinar & Papillary	991(45.7)	914(46.5)	77(37.6)		
Solid & Micropapillary	590(27.2)	477(24.3)	113(55.1)		
TTF-1	2174	1969	205		
Positive	2068(95.1)	1886(95.8)	182(88.8)	9.355E-06^*^	19.639
Negative	106(4.9)	83(4.2)	23(11.2)		
Napsin-A	2174	1969	205		
Positive	2000(92.0)	1823(92.6)	177(86.3)	1.717E-03^*^	9.830
Negative	174(8.0)	146(7.4)	28(13.7)		

Note: ^*^P < 0.05

**Table 6:** Cytological features associated with negative SMARCA2 expression in lung adenocarcinoma

**Factors**	**OR**	**95% Confidence interval**	**P-value**
Nuclear grade			
High	1.000		
Intermediate	0.467	0.338–0.643	3.383E-06^*^
Low	0.408	0.148–0.956	0.056
Histological			
Lepidic	1.000		
Acinar & Papillary	3.285	1.893–6.122	6.447E-05^*^
Solid & Micropapillary	5.331	1.527–16.470	5.019E-03^*^
TTF-1			
Positive	1.000		
Negative	2.427	1.445–3.940	5.024E-04^*^

### 3.4 Survival analysis

**Fig. 3:**
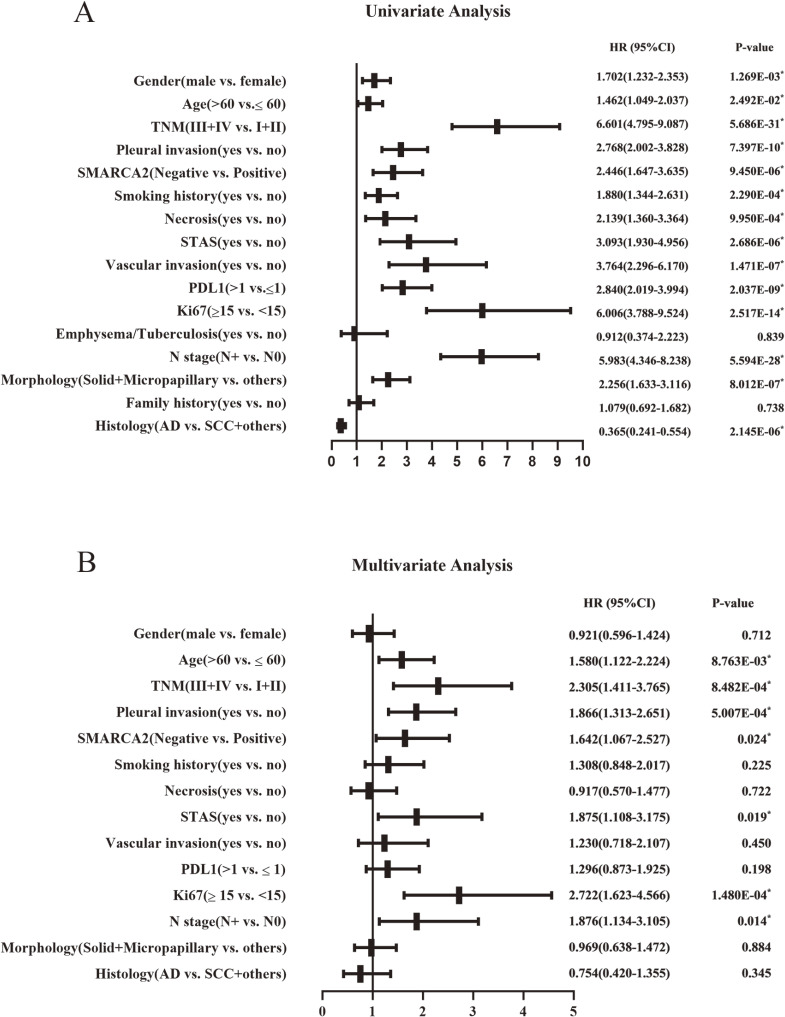
Univariate Analysis Results (**A**) and Multivariate Analysis Results (**B**) of OS in NSCLC. Note: NSCLC, non-small cell lung cancer; STAS, spread through air spaces; AD, adenocarcinoma; SCC, squamous cell carcinoma; HR, hazard ratio; CI, confidence interval; OS, overall survival; *P < 0.05.

**Fig. 4:**
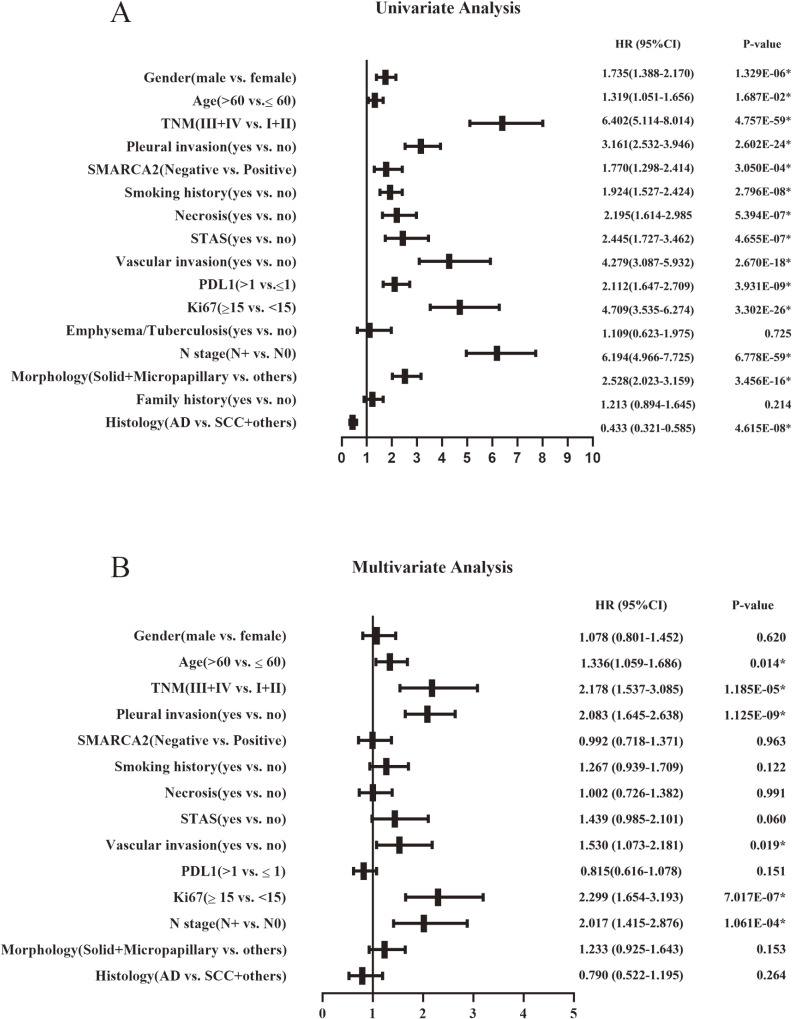
Univariate Analysis Results (**A**) and Multivariate Analysis Results (**B**) of PFS in NSCLC. Note: NSCLC, non-small cell lung cancer; STAS, spread through air spaces; AD, adenocarcinoma; SCC, squamous cell carcinoma; HR, hazard ratio; CI, confidence interval; PFS, progression-free survival; *P < 0.05.

**Fig. 5:**
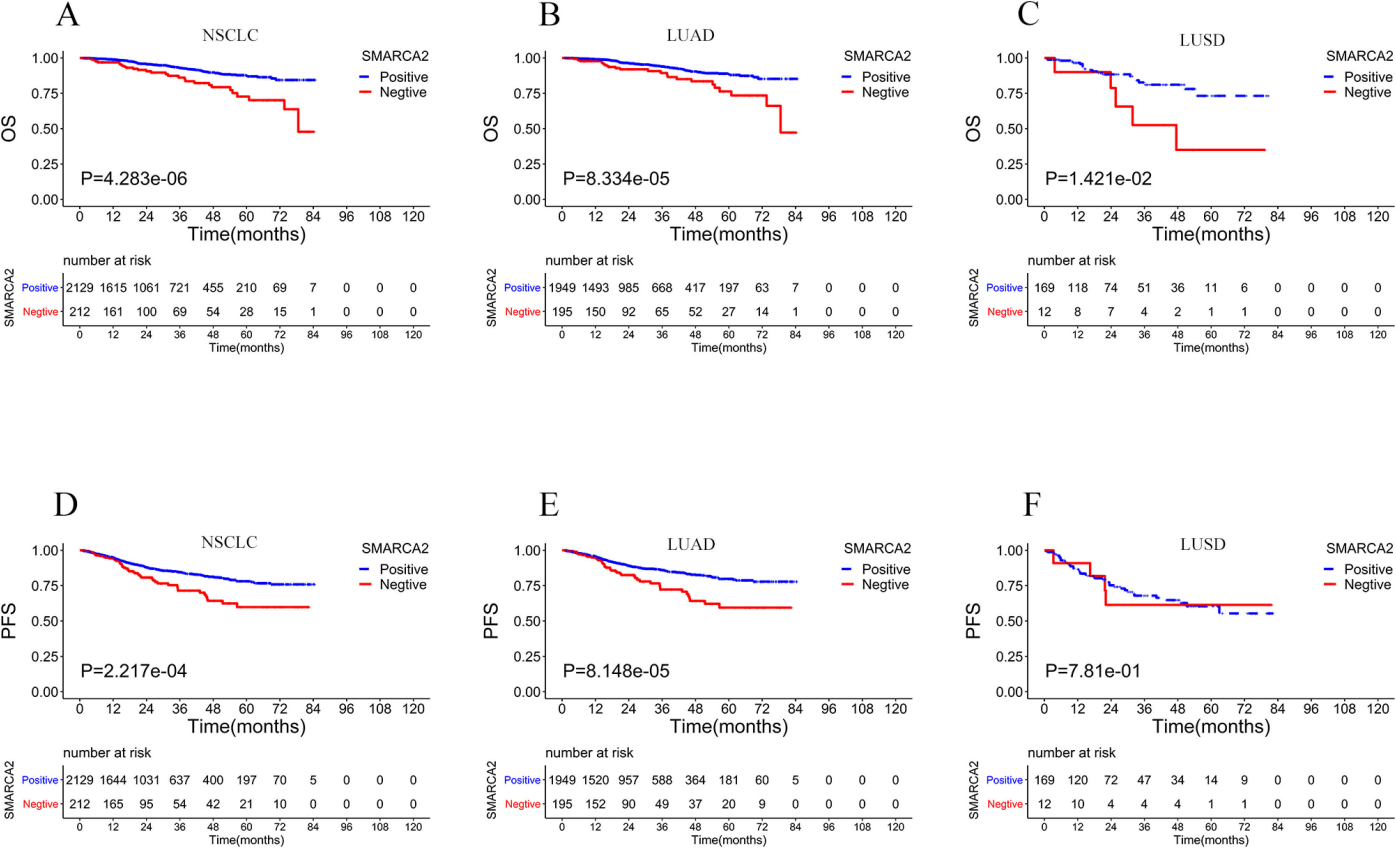
Kaplan-Meier survival curve of OS and PFS for SMARCA2 expression in 2341 cases of NSCLC (**A**, **D**), 2144 cases of LUAD (**B**, **E**), and 181 cases of LUSD (**C**, **F**). Note: LUAD: lung adenocarcinoma; LUSD: lung squamous cell carcinoma.

Among the 2390 NSCLC cases, the cases that were lost to follow-up immediately after discharge from the hospital were deleted. A total of 2341 patients had final follow-up results, and the number of deaths was 151 (6.5%). The median follow-up times of OS and PFS were 23.8 months (range from 1 to 84.7 months) and 23.2 months (range from 1 to 84.5 months), respectively. SMARCA2 expression, sex, age, pathological stage, pleural invasion, smoking history, necrosis, STAS, vascular invasion, PD-L1 expression, Ki67 expression, history of underlying lung disease, lymphatic metastasis, histomorphology, family history, and pathological type were included in the univariate analysis to assess the significance of each variable on OS and PFS. Among all of the variables included above, OS and PFS were shorter in the SMARCA2-negative expression group than in the SMARCA2-positive group in NSCLC. In addition, male sex, age >60 years, III and IV pathological stage, pleural invasion, smoking status, necrosis, STAS, vascular invasion, positive PD-L1 expression, Ki67 ≥ 15%, lymphatic metastasis, morphology with solid and micropapillary components, squamous carcinoma and other pathological types were significantly associated with shorter OS and PFS. To further assess whether SMARCA2-negative expression was an independent predictor of OS and PFS in NSCLC, we then performed a multivariate analysis using the Cox proportional risk regression model, and all variables with p < 0.05 in the univariate analysis were included in the multivariate analysis. The results showed that SMARCA2-negative expression, age > 60 years, III and IV pathological stage, pleural invasion, STAS, Ki67 ≥ 15%, and lymphatic metastasis were independent prognostic factors associated with shorter OS (Fig. 3 A, B). Age >60 years, III and IV pathological stage, pleural invasion, vascular invasion, Ki67 ≥ 15%, and lymphatic metastasis were independent prognostic factors associated with shorter PFS, and SMARCA2 was not an independent prognostic factor for short PFS (Fig. 4 A, B). Kaplan–Meier analysis and log-rank test showed that the SMARCA2-negative group had a shorter OS than the positive group for NSCLC. In both the major subtypes of NSCLC, lung adenocarcinoma and lung squamous cell carcinoma, the SMARCA2-negative group had a worse prognosis than the positive group (Fig. 5 A, B, C). In terms of PFS, the results of the Kaplan–Meier analysis showed that SMARCA2-negative patients had poor PFS for NSCLC and lung adenocarcinoma, but there was no difference between the two groups for lung squamous cancer (Fig. 5 D, E, F).

## 4. Discussion

Loss of the SWI/SNF complex resulted in an increased risk of tumor development in a mouse model study [13]. As a tumor suppressor, loss of subunits from the complex is considered to be a predictor of a poor outcome [12]. Abnormal alterations of SWI/SNF can be found in approximately 20% of human tumors [14]. In our research study, 2390 NSCLC patients were screened on the TMA platform. It is one of the largest platforms to analyze SMARCA2 expression and clinicopathological characteristics of NSCLC. Among these cases, the deletion ratios of subunits (i.e., SAMRCA2, SMARCA4, ARID1A, and ARID1B) in NSCLC are 9.3%, 1.8%, 1.2%, and 0.4%, respectively, with evidence of codeficiency of different subunits. The percentage of subunit-negative patients in our study was generally consistent with a previous report [15, 16]. However, the ARID1A-negative rate was 13.5% in the 171 NSCLC case study conducted by Jang et al. [17]. This difference may be related to the different enrollment methods and interpretation criteria of immunohistochemical results for our nonselective cases. Naito et al. [16] also found codeficiency of SWI/SNF complex subunits in a clinical analysis of 1013 cases of NSCLC. The contribution of these subunits to the dysfunction of the SWI/SNF complex and the underlying mechanism of the development of cancer remain unknown.

In addition, we found, for the first time, the deletion ratios of the subunits in mucinous adenocarcinoma (a variant of lung adenocarcinoma) in a large sample. In previous small sample clinical studies of lung cancer, researchers only described the loss proportion of complex subunits in adenocarcinoma and squamous cell carcinoma and did not systematically describe the type of mucinous adenocarcinoma [17, 18]. In our study, among the 90 mucinous adenocarcinoma cases, the deletion ratios of SMARCA4, SMARCA2, and ARID1A subunits were 6.7%, 5.6%, and 4.5%, respectively. This finding can contribute to the further exploration of the pathogenesis and treatment strategies of mucinous adenocarcinoma.

Matsubara et al. [19] found that loss of SMARCA4 and SMARCA2 correlated with features of a mesenchymal-like phenotype with solid predominant histology in NSCLC. Yoshimoto et al. [20] also found that SMARCA2 staining was significantly lower or negative in the invasive parts in large cell carcinomas. The expression of SMARCA2 in NSCLC was analyzed further in our study, showing the following relatively high risk factors for the occurrence of SMARCA2-negative expression in NSCLC: male, T3 and T4 stage, moderate and poor differentiation, ≥2 cm tumor, adenocarcinoma, Ki67 ≥ 15%, and SOX-2-negative expression. In particular, lesions occurring in the middle lobe of the right lung were found to be a relatively high-risk factor for SMARCA2-negative expression, which has not been clearly reported in the available literature. Our above results may provide further consideration for the clinical screening of SMARCA2-negative NSCLC.

Naito et al. [16] showed that the proportion of pathological stage II, III and IV NSCLC was higher with SWI/SNF loss and that SWI/SNF loss was associated with shorter OS and recurrence-free survival than SWI/SNF intact in stage I NSCLC. In our study, pathological stage was only associated with SMARCA2-negative expression in univariate analysis but not in multifactorial logistic regression analysis. Furthermore, multifactorial logistic regression analysis revealed that T3 and T4 stage independently affected SMARCA2-negative expression. Therefore, T stage might be the main factor influencing SMARCA2-negative expression compared with N stage and M stage.

Nambirajan et al. [21] found that SMARCA4-negative expression tends to present with TTF-1-negative lung adenocarcinomas. In the analysis of different lung cancer cell lines and 93 patients with primary lung adenocarcinoma, Matsubara et al. [19] also found that deletion of SMARCA4 and SMARCA2 was found in lung adenocarcinoma lacking epithelial markers such as TTF-1, CK7, and MUC1. Mechanistically, it has been suggested that both concurrent or nonconcurrent deletion of SMARCA4 and SMARCA2 could further accelerate the process of poor tumor differentiation and EMT. In our study, SMARCA2-negative expression in lung adenocarcinoma was significantly associated with high-grade nuclei, acinar and papillary histological subtypes, and with solid and micropapillary histological subtypes. It has also been shown that the likelihood of SMARCA2-negative expression is 2.427 times higher in TTF-1-negative lung adenocarcinomas than in TTF-1-positive lung adenocarcinomas. The above findings suggest that SMARCA2-negative expression is interrelated with the high invasiveness of tumors and that tumors with SMARCA2-negative expression tend to be poorly differentiated and even accompanied by dedifferentiation and undifferentiation. Moreover, SMARCA2 might be useful as a differential diagnostic indicator in primary lung adenocarcinoma, especially in TTF-1-negative tumors.

In our study, the SMARCA2-negative group had a worse survival and prognosis than the SMARCA2-positive group, consistent with the findings from a previous study [22]. Our data show that SMARCA2-negative expression is an independent prognostic factor for shorter OS. Meanwhile, Kaplan–Meier curve statistical analysis of the results for NSCLC and its two major subtypes, i.e., lung adenocarcinoma and lung squamous cell carcinoma, implies that SMARCA2 is a potential target for therapeutic use and a promising prognostic marker of NSCLC. Our study might provide new insights into the relationship between SMARCA2-negative expression and the clinical outcome of NSCLC.

Agaimy et al. [23] and Tessier-Cloutier et al. [24] found that SWI/SNF subunits were absent in undifferentiated carcinomas, especially those with rhabdoid morphological features, such as those derived from gastrointestinal and pancreatic cancers. Karnezis et al. [25] also found that SWI/SNF subunit deletions occur in differentiated tumors, such as endometrial adenocarcinoma, and SWI/SNF subunit alterations are often secondary or tertiary events of cell cloning, leading to altered cell morphology, altered cell activity, or dedifferentiation. However, in our study, most cases with SMARCA2-negative expression had adenocarcinoma or squamous carcinoma differentiation. Moreover, SMARCA2-negative expression was significantly associated with SOX-2-negative expression, whose presence maintains the stem cell phenotypic characteristics of cancer cells [26]. This might explain the lack of a tumor stem cell phenotypic differentiation direction in SMARCA2-negative NSCLC in our study. In a study of the expression of SOX-2 in two independent cohorts of lung cancer, Velcheti et al. [27] found that overexpression of SOX-2 suggests a good prognosis for NSCLC. The correlation between SMARCA2 and SOX-2 in our results might indirectly suggest that SMARCA2 deletion leads to a poor prognosis of NSCLC. However, the specific mechanism underlying the interaction between SMARCA2 and SOX-2 needs further investigation.

Matsubara et al. [19] showed that SMARCA4 deletion is more involved in the progression of EGFR wild-type lung cancer but not EGFR-mutant tumors, and it was hypothesized that the coexistence of EGFR mutation and SMARCA4 deletion is not suitable for the survival of tumor cells. Herpel et al. [15] also found that SMARCA4 deletion was associated with wild-type EGFR in a clinical case study. However, we found no difference in SMARCA2-negative expression between EGFR mutant and wild-type NSCLC in 569 cases with PCR results for EGFR. Similarly, there were no differences in SMARCA2-negative expression between ALK-positive and ALK-negative cases. These findings indicate that SMARCA2 could be an additional genetic event independent of EGFR mutations and ALK mutations in NSCLC.

Additional studies are required to confirm whether SMARCA2 is a direct driving gene or a passenger gene or whether there are epigenetic regulatory factors. We did not detect any remaining cases without EGFR results, and a statistical deviation could not be completely ruled out. In any case, intratumoral heterogeneity deserves the utmost attention. If a SMARCA2 deletion is found in combination with other driving gene mutations, a combination of different target drugs could be considered for treatment to improve its clinical efficacy.

The research of Liu et al. [28] found that SMARCA2 silencing is driven by SMARCA2 promoter polymorphism, which is considered to be associated with histone deacetylase (HDAC) recruitment. In a preclinical study, Glaros et al. [29] found that an HDAC inhibitor (HDACi) can upregulate SMARCA2 transcript and protein levels in cell lines, suggesting that HDACi may be a treatment for SMARCA2 deletion tumors. In our study, SMARCA2-negative expression was associated with PD-L1-positive expression, suggesting that immunotherapy with immune checkpoint inhibitors (ICIs) may be effective. Previous studies have shown that HDACis can induce apoptosis in tumor cells by inhibiting histone deacetylation and keeping DNA open for transcription [30]. Blaszczak et al. [31] and McCaw et al. [32] have also verified that HDACis can upregulate tumor antigen expression, increase the number and cytotoxicity of NK and CD8+ cells to enhance cellular immunity, and thus increase the antitumor effects in vivo and in vitro, suggesting that there will be a synergistic effect if they are combined in antitumor therapy. For the application of HDACis in combination with ICIs in antitumor therapy, researchers have conducted preliminary studies in soft tissue sarcoma [33] and lymphoma [34] and achieved good results. At present, there are no studies of HDACis combined with ICIs therapy in lung cancer. Our above findings may provide an approach to the treatment of patients with SMARCA2-negative NSCLC.

## 5. Conclusion

In conclusion, we evaluated an immunohistochemical assay to detect the negative expression of five subunits of SWI/SNF in NSCLC on the TMA platform, focusing on the clinicopathological relevance and prognostic significance of SMARCA2-negative expression in NSCLC. SMARCA2-negative expression is an independent predictor of OS in NSCLC and is a potential target for NSCLC therapy. Our present findings may provide a possibility for exploring SMARCA2 as a clinically transformable indicator in the diagnosis and treatment of NSCLC. However, the exact roles and specific mechanism of SMARCA2 in NSCLC are not yet clear; therefore, more functional studies and in vivo validation are required to understand the contribution and mechanism of SMARCA2 in NSCLC.

## Abbreviations

SMARCA2: SWI/SNF Related, Matrix Associated, Actin Dependent Regulator of Chromatin, Subfamily A, Member 2
SWI/SNF: switch-sucrose nonfermenting complex
NSCLC: Non-small-cell Lung Cancer
TMA: Tissue Microarrays
AD: adenocarcinoma
SCC: squamous cell carcinoma
LCC: large cell carcinoma
STAS: spread through air spaces
WT: Wild type
MT: Mutant type
OS: overall survival
PFS: progression-free survival
LUAD: Lung adenocarcinoma
LUSD: Lung squamous cell carcinoma

## Declarations

### Availability of data and materials

All data generated or analyzed during this study are included in this article.
